# Supplementary material for: Impact of Non-Pharmaceutical Interventions on the Incidence and Treatment of Chronic Rhinosinusitis during the COVID-19 Pandemic: A Nationwide Retrospective Cohort Study
Source: J Clin Med. 2023 Oct 19;12(20):6629. doi: 10.3390/jcm12206629 (PMC10607855; doi:10.3390/jcm12206629)
Supplement: Supplementary file 1 [file jcm-12-06629-s001.zip › jcm-2644581-supplementary.pdf]

**Supplementary Materials**

# Impact of Non-Pharmaceutical Interventions on the Incidence and Treatment of Chronic Rhinosinusitis during the COVID-19 Pandemic: A Nationwide Retrospective Cohort Study

**Supplementary Table S1.** Ingredients of antibiotics and steroids

**Supplementary Table S2.** Main NPIs implemented against COVID-19 during the study period in South Korea

**Supplementary Table S3.** Subgroup analysis according to age

**Supplementary Table S1. Ingredients of antibiotics and steroids**

| | Ingredient |
|---|---|
| Antibiotics | flomoxef, cefditoren, cefpodoxime, cefdinir, cefcapene, cefaclor, cefadroxil, cefazolin, cefbuperazone, cefepime, cefetamet, cefixime, cefmetazole, cefminox, cefodizime, cefoperazone, cefotaxime, cefotetan, cefotiam, cefpiramide, cefpirome, cefprozil, cefradine, cefroxadine, ceftazidime, ceftezole, ceftizoxime, ceftriaxone, cefuroxime, cephalexin, cephazedone, cetirizine, cilastatin, nafcillin, amikacin sulfate, amoxicillin, amoxicillin + clavulanate potassium, amoxicillin + sulbactam, sultamicillin, piperacillin + sulbactam, piperacillin + tazobactam, clarithromycin, erythromycin, roxithromycin, levofloxacin, balofloxacin, ciprofloxacin, levofloxacin, moxifloxacin, gemifloxacin, ofloxacin, tosufloxacin, netilmicin, lomefloxacin, arbekacin, ribostamycin, tosufloxacin, kanamycin, fosfomycin, clindamycin, colistin, doxycycline, tetracycline, tigecycline, minocycline, gentamicin, isepamicin, meropenem, ertapenem, lincomycin, vancomycin, linezolid, metronidazole, trimethoprim + sulfamethoxazole |
| Systemic or topical steroid | methylprednisolone, prednisolone, dexamethasone, mometasone, fluticasone, dexamethasone, ciclesonide |

**Supplementary Table S2. Main NPIs implemented against COVID-19 during the study period in South Korea**

| Non-pharmaceutical interventions | Start and end dates |
|---|---|
| **Wearing facial masks** | |
| Five-day rotation facial masks distribution system | 2020/03/06 to 2020/06/18 |
| Mandatory in all spaces | 2020/02/23 to 2020/05/25 |
| Obligation at public transport | 2020/05/26 to 2021/12/31 |
| Obligation in all spaces | 2020/11/13 to 2021/12/31 |
| **Social Distancing** | |
| Social distancing campaign | 2020/03 |
| Enhanced social distancing steps* | 2020/03/21 to 2021/12/31 |
| **Registration for visiting public space (KI-Pass QR code check-in)** | |
| Vaccination certificate or COVID-19 polymerase chain reaction negative certificate | 2020/06/01 to 2021/12/31 |
| **Closure of educational institutions** | |
| Total closure of all educational institutions (except emergency daycare) | 2020/02/23 to 2020/05/20 |
| On-line classes | 2020/04/09 to 2020/06/07 |
| Partial closure of secondary schools | 2020/05/20 to 2020/06/07 |
| Partial closure of primary schools | 2020/05/27 to 2020/06/07 |

*Restrictions on private gathering, multi-use facilities, event gathering, watching sports, wedding hall, and religious activities

## Supplementary Table S3. Subgroup analysis according to age

| Parameter | Patients aged 0–19 | | | Patients aged 20–39 | | | Patients aged 40–59 | | | Patients aged 60 or older | | |
|---|---|---|---|---|---|---|---|---|---|---|---|---|
| | Pre-NPI | NPI | P-value | Pre-NPI | NPI | P-value | Pre-NPI | NPI | P-value | Pre-NPI | NPI | P-value |
| Monthly age- and sex- specific incidence rates* | 29.09 ± 8.56 | 8.80 ± 2.97 | < 0.001 | 30.16 ± 7.57 | 10.44 ± 2.41 | < 0.001 | 36.38 ± 10.71 | 12.81 ± 3.11 | < 0.001 | 49.65 ± 14.24 | 19.73 ± 4.38 | < 0.001 |
| Monthly number of total outpatients | 14803.4 ± 4464.9 | 9151.9 ± 2493.7 | < 0.001 | 17810.6 ± 4235.9 | 13916.4 ± 1791.9 | < 0.001 | 29555.5 ± 6313.6 | 23864.7 ± 2657.7 | < 0.001 | 34851.6 ± 7518.8 | 30734.9± 3004.5 | 0.017 |
| Annual frequency of outpatient visits per patient | 7.91 ± 20.20 | 2.64 ± 10.77 | < 0.001 | 7.84 ± 25.15 | 2.61 ± 11.11 | < 0.001 | 8.74 ± 26.47 | 2.92 ± 11.08 | < 0.001 | 9.58 ± 29.21 | 3.41 ± 12.91 | < 0.001 |
| Monthly number of total patients hospitalized | 220.5 ± 56.58 | 126.4 ± 33.15 | < 0.001 | 507.5 ± 93.31 | 435.3 ± 46.61 | 0.002 | 923.0 ± 153.9 | 798.3 ± 73.98 | < 0.001 | 896.4 ± 171.8 | 822.8 ± 70.0 | 0.057 |
| Annual frequency of hospitalizations per patient | 0.13 ± 2.36 | 0.04 ± 0.89 | < 0.001 | 0.19 ± 2.38 | 0.09 ± 0.75 | < 0.001 | 0.26 ± 3.66 | 0.11 ± 0.91 | < 0.001 | 0.27 ± 4.10 | 0.12 ± 2.04 | < 0.001 |
| Monthly number of total patients who underwent surgery | 92.92 ± 40.23 | 73.17 ± 21.97 | 0.040 | 333.80 ± 66.61 | 294.50 ± 27.89 | 0.011 | 657.40 ± 131.70 | 601.4 ± 54.45 | 0.060 | 512.4 ± 113.8 | 538.8 ± 58.0 | 0.311 |
| Annual frequency of surgeries per patient | 0.05 ± 1.64 | 0.02 ± 0.50 | < 0.001 | 0.11 ± 1.33 | 0.06 ± 0.50 | < 0.001 | 0.16 ± 2.25 | 0.09 ± 0.78 | < 0.001 | 0.12 ± 1.73 | 0.07 ± 0.74 | < 0.001 |
| Monthly number of total patients prescribed antibiotics | 10946.6 ± 3479.8 | 6234.0 ± 2164.9 | < 0.001 | 11854.0 ± 2952.1 | 7969.7 ± 1525.9 | < 0.001 | 18374.8 ± 4095.7 | 12389.1 ± 2216.6 | < 0.001 | 19871.3 ± 4425.1 | 14289.0 ± 2365.1 | < 0.001 |
| Annual frequency of antibiotics prescriptions per patient | 5.88 ± 17.10 | 1.86 ± 8.52 | < 0.001 | 5.32 ± 20.54 | 1.54 ± 7.82 | < 0.001 | 5.74 ± 21.70 | 1.56 ± 7.53 | < 0.001 | 5.86 ± 22.88 | 1.72 ± 8.71 | < 0.001 |
| Monthly number of total patients prescribed steroid | 2701.8 ± 799.9 | 2053.3 ± 453.9 | 0.001 | 5217.7 ± 1342.1 | 4607.8 ± 575.9 | 0.046 | 7350.7 ± 1632.4 | 6430.3 ± 613.1 | 0.013 | 5991.3 ± 1339.0 | 5603.0 ± 491.5 | 0.186 |
| Annual frequency of steroid prescriptions per patient | 1.55 ± 9.19 | 0.57 ± 3.74 | < 0.001 | 2.30 ± 12.94 | 0.84 ± 4.47 | < 0.001 | 2.11 ± 11.84 | 0.78 ± 4.70 | < 0.001 | 1.67 ± 11.40 | 0.63 ± 4.18 | < 0.001 |

*per 100,000 population

Data are presented as number (%) or mean ± standard deviation
